# A randomised pilot equivalence trial to evaluate diamagnetically enhanced transdermal delivery of key ground substance components in comparison to an established transdermal non-steroidal anti-inflammatory formulation in males with prior knee injury

**DOI:** 10.1371/journal.pone.0211999

**Published:** 2019-02-22

**Authors:** Bill Vicenzino, Peter Lawrenson, Asaduzzaman Khan, Aiofe Stephenson, Luke Heales, Heather A. E. Benson, Anthony Wright

**Affiliations:** 1 School of Health and Rehabilitation Sciences, University of Queensland, Brisbane, Queensland, Australia; 2 School of Pharmacy and Biomedical Science, Curtin University, Perth, Western Australia, Australia; 3 School of Physiotherapy and Exercise Science, Curtin University, Perth, Western Australia, Australia; MD Anderson Cancer Center, UNITED STATES

## Abstract

**Objective:**

This pilot study assessed the efficacy of a knee guard device, which used magnetophoresis to transdermally deliver Glucosamine, Chondroitin and Hyaluronic Acid in a cohort of individuals with prior knee injury. The aim was to determine if the change in physical function and pain with the knee guard device was equivalent to the change produced by an established topical NSAID formulation containing diclofenac sodium 1%.

**Methods:**

A randomized, controlled, equivalence trial evaluated outcomes following treatment with the knee guard device or NSAID formulation. The study recruited 114 male participants (aged 40–55 years). Participants were randomly allocated to wear the knee guard device or to use a NSAID gel daily for two weeks. The primary outcomes were the knee injury osteoarthritis function score (KOOS-F) and an aggregated function score (AFS). The lower extremity functional scale (LEFS), pain numerical rating scale (PNRS), global rating of change (GROC) and other KOOS scores were also evaluated.

**Results:**

Multiple linear regression analyses indicated that there were no significant differences between the interventions for changes in the primary outcomes of AFS and KOOS_F. The 95% confidence interval (-2.89 to 5.15) of the estimated treatment difference for KOOS-F was within the lower (-5.61) and upper (5.61) bounds of the 7% equivalence margin for that measure, The mean value for the AFS was within, but the 95% CI (-3.11 to 7.37) exceeded the 7% equivalence margin (-2.97 to 2.97) for that measure. There was a significant difference in PNRS, which favored the knee guard device.

**Conclusion:**

The knee guard device demonstrated equivalence for the KOOS-F measure but not the AFS measure of function over the two week trial period when compared to a widely available NSAID gel that has been shown to be superior to placebo. The knee guard produced a greater reduction in pain report (p = 0.002) than the NSAID gel. Users of the knee guard device experienced more skin irritation than participants using the NSAID gel. Further research is required to fully evaluate the therapeutic potential of this innovative treatment approach.

## Introduction

Osteoarthritis (OA) affects more people than any other joint disease and is a major source of disability in developed countries [1, 2]. Knee OA is the second most common form of osteoarthritis with the prevalence ranging from 18–28% in over 55s in the United Kingdom [1]. Knee injury appears to be an independent risk factor, particularly for unilateral knee OA [3] and those with a documented history of knee injury have a greater than 5 fold increased risk of developing knee OA [4]. The cohort with significant prior knee injury is therefore a key group to target for interventions to prevent or limit the development of OA.

Given the steadily increasing economic and societal burden of OA there is an increased focus on strategies to manage knee injuries and the development of OA. The first line of management for knee OA includes regular exercise and bodyweight reduction [5, 6] with the second line being the use of medical/pharmaceutical agents such as simple analgesics, non-steroidal anti-inflammatory drugs (NSAIDs) and medications such as duloxetine [7]. NSAIDs, particularly oral dosage forms, are normally only used in patients with a relatively low risk profile for those medications [7].

The use of compounds targeted to the synovial joint (hyaluronic acid) and articular cartilage (chondroitin and glucosamine) has emerged as an alternative to the symptomatic second line strategies. Intra-articular injection of hyaluronic acid to maintain synovial fluid function has been a treatment option for several years [8]. It remains controversial despite a systematic review of meta-analyses [9] and expert consensus [10] indicating that it is a valuable treatment for early knee OA. Chondroitin and glucosamine exert a beneficial effect on the metabolism of different cell lines that are involved in the development of OA [11, 12]. They increase type II collagen and proteoglycan synthesis in human articular chondrocytes, reduce the production of some pro-inflammatory factors and proteases and improve the anabolic/catabolic balance of the extracellular cartilage matrix [11, 12]. Clinical trials have reported some beneficial effects of oral chondroitin and glucosamine with a small but significant reduction in the rate of decline in joint space width and some symptomatic improvement [11]. These benefits have been supported by international guidelines [13]. Concerns have been expressed however in relation to the bioavailability of chondroitin and glucosamine due to extensive first pass metabolism following oral administration [14].

Delivering hyaluronic acid, chondroitin and glucosamine transdermally could provide improved bioavailability compared to oral administration, but they have physicochemical characteristics that are unfavorable for skin permeation [15–18]. Advanced delivery systems are required to enhance their transdermal penetration. Magnetophoresis is such a delivery technology that has been shown to enhance the skin penetration of drug and peptide molecules and 20nm gold particles [19–22]. The knee guard device (OBJ Pty Ltd, Perth) is a patented wearable knee support that uses magnetophoresis to enhance transdermal delivery of therapeutic compounds from topical applications beyond that of passive diffusion [23]. It has yet to be tested as a treatment intervention in a human population, thus the reason for this pilot trial.

The knee guard device is applied to the anterior aspect of the knee joint. It contains replaceable gel pods which release the Lubricen formulation with diamagnetic backing sheets that enhance the transdermal permeation of gel constituents.

This pilot study assessed the efficacy of the knee guard device, which uses magnetophoresis technology, in a cohort of individuals with prior knee injury who are at increased risk of developing OA [4]. The main aim of the study was to determine if the change in physical function and pain with the knee guard device was equivalent to the change produced by an established NSAID formulation containing diclofenac sodium 1%.

## Materials and methods

A randomized, controlled, pilot equivalence trial design was used to evaluate outcome following treatment with the knee guard device or the NSAID formulation in middle-aged males following knee injury. The trial was approved by the Medical Research Ethics Committee at the University of Queensland (MREC #2014001527, 2^nd^ February 2015) and the trial protocol was posted on the Australia New Zealand Clinical Trials Register (ANZCTRN12615000345583).

### Participants

The study recruited male participants aged between 40 and 55 years who were in good general health and currently participating in regular physical activity (>2 hours per week), despite a prior history of knee injury requiring arthroscopy surgery, and/or a prolonged history of recurrent knee pain (>2/10 on pain numerical rating scale). Key exclusion criteria included: patellofemoral joint dysfunction, significant ligamentous deficiency and a history of cardiac disease, high blood pressure, asthma or diabetes.

Following initial expression of interest from volunteers, a preliminary telephone interview was used to determine the participant’s general suitability for the study and to arrange an appropriate time for testing. Screening questionnaires were completed by email and testing was carried out at the St Lucia Campus of the University of Queensland.

Participants completed the Fitness Australia Adult Pre-Exercise Screening tool to determine their suitability for participation in the study [24]. Participants who met the inclusion criteria, had no exclusion criteria and were classified as low risk on the Pre-Exercise Screening Tool were asked to provide written informed consent prior to participating in the study.

### Interventions

Participants were allocated to wear the knee guard device (OBJ Pty Ltd, Perth) or to use a NSAID gel. The knee guard device incorporated a magnetophoretic backing and gel filled pods (1 g gel) including Lubricen (a Therapeutic Goods Administration (TGA) approved formulation including Glucosamine Sulphate 10mg, Chondroitin Sulphate 2.5mg, Hyaluronic Acid 2.5mg and Menthol 40mg per gram). Participants were required to wear the knee guard device for a period of 3–5 hours per day.

The NSAID gel was a commercially available formulation of diclofenac sodium (1%) that was applied 4 times daily, following manufacturer’s recommendations. Participants were asked to apply 4 g of the gel 4 times daily and they were provided with a card which guided them to apply a standard amount each time [25]. The diclofenac gel is a well-established symptomatic treatment with pooled data from three randomized controlled trials demonstrating that the diclofenac sodium gel formulation achieves superior outcomes in terms of pain, function and pain on movement when compared to placebo formulations in patients with knee OA [26]. The difference between active treatment and vehicle control indicated small to medium effect sizes for WOMAC pain (0.22), WOMAC function (0.25) and VAS rating of pain on movement (0.205) [27]. A recent Cochrane review concluded that diclofenac was significantly more effective than vehicle for reducing OA pain with a number need to treat (NNT) of 9.8 (CI 7.1–16) for a 50% reduction in pain [28]. The gel was provided in plain tubes without manufacturer logos. The duration of treatment was limited to two weeks in order to comply with the standard clinical recommendations not to apply the NSAID gel on a daily basis for more than two weeks.

### Randomization and masking

Participants were randomly assigned (computer generated random assignment by an investigator not involved in recruitment or data collection/analysis) on a 1:1 basis to either wear the knee guard device or to use the diclofenac sodium (1%) gel over a two week period. A research assistant who was not involved in the baseline screening or any of the outcome measurements administered the randomized allocation and provided the knee guard device or diclofenac sodium (1%) gel to the participant as per the randomization schedule. The research assistant who took the physical outcome measures was blind to the allocated treatment. The participants were not blind to their allocated intervention, but were instructed to not tell the research assistant taking the physical measures which treatment they were using (reminded by the other research assistant on arrival at the testing site). To facilitate the blinding of the research assistant taking the physical outcome measures, the participant also wore a sleeve over both knees at baseline and follow up.

### Outcome measures

The primary and secondary outcomes were taken at baseline and the primary end point of 2 weeks. Baseline measures of height and weight were also obtained.

The primary outcomes were the knee injury osteoarthritis score (KOOS) function score and an aggregated function score (AFS). Pain and function was evaluated using the KOOS [29], which assesses a participant’s opinion about their knee and associated problems. It is intended for use with knee injury that can result in post-traumatic OA. It consists of 5 subscales; pain (KOOS-P), other symptoms (KOOS-S), function in daily living (KOOS-F), function in sport (KOOS-SP) and knee related quality of life (KOOS-Qol). The 5 subscales are reported separately as a percentage score, with 0 representing extreme problems and 100 representing no problems. It has high test retest reliability (ICC >0.75) [30]. The test was administered in a paper and pencil format.

The aggregated function score was determined as the total time in seconds required to complete: (a) 10 m shuttle run, (b) modified Balsolm agility run, (c) stair climb, and (d) 20 step down-up. For the 10 m shuttle run, two marks were placed on a straight runway 10 m apart. Two 0.5 kg weight bags were placed at the 10 m mark. The participant was asked to run as fast as possible from the starting line to the 10 m mark, pick up a bag, return the bag to the ground behind the starting line, run back to the 10 m mark, pick up the 2nd bag and bring it back to the start line. The modified Balsom agility run requires the participant to negotiate a 12 m course with 5 gates that required two changes of direction and two turns, as rapidly as possible. The original Balsolm agility test was 15m in length, which was reduced in this study due to space restriction for safe execution of the test. The stair climb required the participant to descend and ascend a flight of 20 stairs, with a landing half way down and at the bottom of the stairs. The participant was required to touch every step and to move as fast as possible without using the hand rail or any support. The step down-up test required the participant to step down and up from a 15 cm block 20 times using the affected limb while keeping the unaffected knee fully extended. The sole and heel of the unaffected leg had to touch the ground and the top of the block each time. All these tests were repeated twice with a 3 min rest interval and the best time recorded. To minimize risk of injury all participants warmed up by completing 5 min stationary cycling at 11–13 RPE followed by 5 min of dynamic mobility drills that imitated the changes of direction and physical demands required during the physical function tests.

The secondary outcomes were the additional KOOS measures, the lower extremity functional scale (LEFS) and a pain numerical rating scale (PNRS). The LEFS is a 20-item questionnaire with responses scored on a 5 point scale where 0 was unable to do/extremely difficult and 4 was no difficulty. The maximum score is 80 and 9 has been calculated as a minimum level of detectable change [31]. The PNRS is an 11 point scale with 0 being no pain and 10 being worse pain imaginable. Participants were asked to rate their worst level of pain as a whole number over the past week.

A global rating of change score (GROC), compliance and adverse events with treatment, as well as satisfaction with treatment were collected at 2 weeks. Participants rated how their condition had changed from baseline to follow up on a 7 point GROC scale where 1 corresponded to much worse, 7 to much better and 4 was no change (or the same). The GROC was dichotomized with scores greater than 4 representing an improvement (i.e., 5 a little better, 6 better or 7 much better) and scores less than 5 being no change or worse.

Participants completed study diaries, in which they answered questions on compliance to treatment, satisfaction with their knee, and on adverse events. The compliance question asked the participant to rate compliance on a 5 point scale, where 4 was full compliance, 3 was mostly compliant (>75% of treatment accomplished), 2 was complied with at least 50% of treatment, 1 was complied with less than 50% and 0 was not compliant at all (did not use treatment). Satisfaction was rated by asking participants to answer yes or no to the question, ‘Is your current level of pain satisfactory when you consider your level of activity and general function?’

Adverse events were determined by asking participants each week (by phone when they were not physically being tested) if they had encountered any issue (yes or no) during the prior week intervention period. If the participant indicated yes they were asked to comment/describe further on the nature of the issue. The comments were then categorized on a 4 point scale according to severity, with 3 indicating skin breakdown (e.g., blistering, raw, breakdown of the skin etc.), 2 indicating minor changes in the skin condition (e.g., dry, flaky, red or rash), 1 indicating symptoms of altered sensation (e.g., itchy, heat, tingling) and 0 indicating no reaction at all.

### Data analysis

It was determined a priori (G*Power, Heinrich Hein University, Dusseldorf, Germany) using a simple pairwise comparison (t-test) that a sample size of 114 participants would be required to determine a pair-wise 7% equivalence rate on the KOOS-F (physical function) with an 11% Standard Deviation (SD) assuming 90% power, 95% confidence interval (two sided) and a 10% drop out rate [26].

We used Stata 14/SE (StatCorp, College Station, TX) to conduct the statistical analysis. Statistical analyses between groups were performed using intention-to-treat. Prior to conducting any analysis, the collected data were examined for missing values. Collinearity of the covariates (potential confounders) was assessed before including them in the multivariable modelling for testing the hypotheses. To examine whether the change (measured using a pre-post change score) in primary and secondary outcomes between the two interventions were comparable, multiple linear regression modeling was used. This modeling compared the equivalence of the two interventions, adjusted for the effects of baseline function and other potential confounders. Potential confounders included BMI and pre-existing pain levels, as they are related to functional impairment (the primary outcome) [32].

The AFS was reduced to the aggregate of 3 tests: 10 m shuttle, Balsom agility run and stair climb, because the step down-up was unable to be completed by 20 participants (due to pain).

An alpha level of 0.05 was considered significant in the final modelling. The group data are presented as means and standard deviations, while the between group differences are presented in the form of regression coefficients and their 95% confidence intervals as derived from the regression models. The residuals of the fitted models were examined to ensure that all required assumptions were met. In the event of non-normally distributed residuals, appropriate transformation was implemented on the outcome variables in order to implement appropriate parametric statistical analyses.

## Results

**Fig 1:**
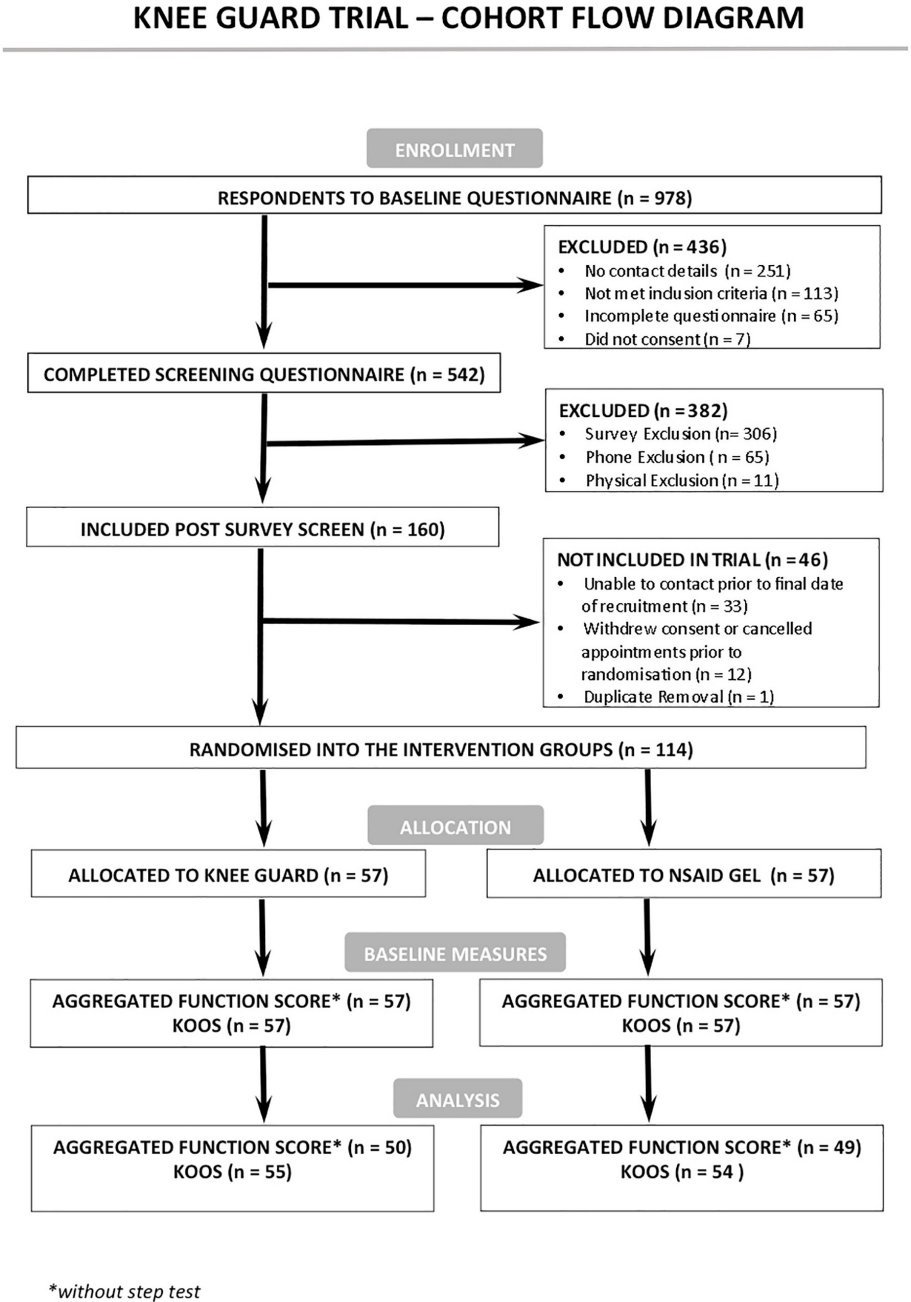
Knee guard trial cohort flow diagram.

One hundred and fourteen male participants enrolled between 113^th^ July and 4^th^ December 2015, with the last follow up on the 22^nd^ of December 2015. Participants were recruited through a media campaign (Facebook, magazines, newspaper, radio advertisements) in the Brisbane metropolitan area during 2015. The participant flow chart shows the respondents to the advertising campaign, the reasons for exclusion at the various stages, numbers allocated and then numbers followed up (Fig 1). All participants completed the AFS and KOOS at baseline while 99 and 109 completed the follow-up assessments with attrition rates being 13% and 4% respectively. The loss to follow up was equally distributed between the groups (i.e., AFS 7/57 & 8/57 for the knee guard device and NSAID gel respectively; likewise 2/57 & 3/57 for KOOS). The median follow up time was 14 days (IQR: 14 to 16). The two groups were comparable at baseline for age (mean (SD): 47.7 (4.4) and 48.4 (4.8) years) and BMI (28.2 (3.8) and 29 (3.5) kg/m^2^).

### Primary outcomes

Multiple linear regression analyses indicated that there were no significant differences between the interventions for changes in the primary outcomes KOOS-F (p = 0.58) and AFS (p = 0.43), The between group differences and their confidence intervals (Table 1), when corrected for BMI and baseline pain intensity, are near null with confidence intervals containing 0, which infers equivalence between the two treatments.

**Table 1:** Group mean (SD) outcome data and between groups mean differences (95% CI, p value, sample size) unless otherwise stated.

Measure`	Knee guard		NSAID		Mean diff (95% CI)	p-value
	Baseline	2 weeks	Baseline	2 weeks		
Primary						
KOOS-F	76.71 (13.16)	82.18 (15.10)	77.80 (16.47)	80.17 (17.83)	1.13 (-2.89 to 5.15)	0.58
AFS3 (sec)	41.81 (8.25)	40.87 (8.95)	42.75 (10.83)	42.39 (11.46)	-2.13 (-3.11 to 7.37)	0.43
Secondary						
KOOS-P	66.47 (12.17)	72.17 (15.71)	66.37 (15.83)	69.90 (17.43)	3.18 (-0.91 to 7.27)	0.13
KOOS-S	64.60 (17.18)	68.17 (20.57)	65.66 (17.90)	68.45 (17.19)	1.28 (-4.57 to 7.13)	0.67
KOOS-Sp	53.96 (18.99)	62.80 (23.20)	48.20 (17.54)	55.93 (25.40)	-0.29 (-3.89 to 3.30)	0.87
KOOS-QoL	41.71 (17.77)	48.09 (20.16)	44.69 (18.71)	49.81 (18.46)	0.18 (-4.24 to 4.61)	0.93
LEFS	60.06 (11.22)	61.68 (13.81)	57.96 (12.07)	59.94 13.83)	-0.55 (-4.32 to 3.22)	0.77
PNRS	5.78 (1.73)	3.91 (2.05)	5.65 (2.17)	4.69 (2.51)	-1.16 (-1.88 to -0.44)	0.002
GROC		38 (69%)		32 (59%)		0.28

`Knee Injury Osteoarthritis Score (KOOS): function in daily living (KOOS-F), pain (KOOS-P), other symptoms (KOOS-S), function in sport (KOOS-SP), knee related quality of life (KOOS-QoL), Lower extremity function scale (LEFS), pain numerical rating scale (PNRS), global rating of change expressed as number improved (%) (GROC).

In order to further evaluate equivalence we calculated the 7% equivalence rate on the KOOS-F from the NSAID group and its 95% confidence interval. The equivalence margin was computed as -5.61 and 5.61. Against this we compared the 95% CI of the intervention group. The 95% confidence interval (-2.89 to 5.15) of the estimated treatment difference for KOOS-F between treatments is within the lower (-5.61) and upper (5.61) bounds of the equivalence margin, which suggests that the two treatments are comparable. The 7% equivalence margin for the AFS from the NSAID group was -2.97 to 2.97. The mean difference was 2.13, which sits within this equivalence margin. However, the CI for the estimated treatment difference for AFS between treatments was -3.11 to 7.37, which exceeds the equivalence margin, indicating considerable variability in the AFS response.

### Secondary outcomes

Multiple linear regression analyses indicated that there were no significant differences between the interventions for the changes in KOOS-P (p = 0.13), KOOS-S (p = 0.67), KOOS-SP (p = 0.87), and KOOS–QoL (p = 0.93). LEFS (β = -0.55, 95%CI: -4.32 to 3.22 p = 0.77) and the GROC score (β = -0.13, 95%CI: -0.43 to 0.18, p = 0.42). The GROC score revealed improvement at follow up in 38 (69%) of the knee guard group and 32 (59%) of the NSAID gel group (Table 1). Chi-squared analysis indicated that there was no difference in the percentage of participants who were improved with each intervention (p = 0.28).

There was a significant difference in PNRS, which favored the knee guard device (β = -1.16, 95%CI: 0.44 to 1.88 p = 0.002). At follow up, the knee guard device group mean (SD) was 3.91 (2.05) on the PNRS compared to 4.69 (2.51) for diclofenac sodium (1%) gel.

Participants self-report of compliance was not different between treatments with 52 (96%) in each group indicating that they had completed at least 50% of the allocated treatment, but only 25 (46%) and 16 (30%) in the knee guard device and NSAID gel groups respectively reporting full compliance. Given that full compliance required participants to wear the knee guard device for 5 hours each day and apply the NSAID gel 4 times each day the overall level of compliance was considered satisfactory.

There was no significant difference in overall satisfaction between treatments with 33 (60%) and 34 (64%) of the knee guard and NSAID gel groups reporting that they were satisfied.

### Adverse events during the treatment period

Skin breakdown (13, 24%) and skin irritation (28, 51%) were present in significantly more (p<0.05) of those who wore the knee guard device, with only 1 (2%) of the NSAID gel group reporting skin break down (Table 2, Chi-square test).

**Table 2:** Adverse events during the treatment period.

Skin reaction	Knee guard device	NSAID gel
Skin breakdown (e.g., blistering)	13 (23.6%)	1 (2%)
Minor skin irritation (e.g., dry, flaky, red or rash)	28 (50.9%)	0
Altered sensation (e.g., itchy, heat, tingling)	1 (1.8%)	0
None	13 (23.6%)	53 (98%)

## Discussion

The knee guard device demonstrated equivalence for the KOOS function score over the two week trial period when compared to a widely available NSAID gel that has been shown to be superior to placebo [26, 27]. The finding of equivalence was demonstrated by the 95% confidence interval of the estimated treatment difference for KOOS-F being within the lower and upper bounds of the equivalence margin for that measure, Equivalence was not demonstrated for the AFS measure, primarily due to the large variability in the AFS response. The finding of equivalence for the KOOS-F measure was further supported by small non-significant between group differences across all outcome measures. This suggests that the device provides therapeutic improvements in function and pain that are potentially valuable for overweight (by BMI), middle-aged males with a prior knee injury and a moderate level of pain (~6/10).

The observed effects occurred with 46% and 30% of the knee guard device and NSAID gel groups, respectively completing all allocated treatment, although the majority of both groups completed at least 50% of the allocated treatment. With approximately 60% of participants indicating overall satisfaction with allocated treatment, there are grounds for further research on optimal dosing regimens.

Treatments like the knee guard device and the NSAID gel are generally regarded as second line treatments, but they are also useful in facilitating individuals to undertake first line treatments such as specific exercise and physical activity. Over the short trial period of 2-weeks of use of the knee guard device, there were positive changes on most of the outcomes. The total AFS time improved by approximately 7 seconds (9.5%) and while there is no established clinically meaningful difference, an improvement of this magnitude supports further consideration as a physical activity adjunct. The recommended minimally important clinical difference of 8–9 for the KOOS [33] was met by the KOOS-SP, which might be an indication that the knee guard device will enable participation in physical activities that have a front line role in managing early onset OA. The other scales of the KOOS did not reach a clinically meaningful improvement, but showed tendencies towards improvement. This was not unanticipated as the dimensions captured by the KOOS are more likely to show change over a longer treatment period. The secondary outcome of worst pain experienced in the previous week changed by a clinically meaningful difference (1.88 points on an 11 point PNRS) in the knee guard device group [34]. This was significantly greater than the change in PNRS for the NSAID gel group.

The current study was the first for this device in humans and was limited to a 2-week intervention period, primarily because that was the recommended maximum daily treatment period for the NSAID gel. Whilst this study focused on short-term outcomes in terms of pain and function the active components in the Lubricen formulation have the potential to positively influence the development of OA. The results of this pilot study indicate that there would be value in conducting prospective studies to determine if regular exercise in combination with wearing the knee guard device was of benefit in delaying the onset of OA in this at risk population.

A limitation of the study is that it was restricted to male participants within a tight age range. The population chosen was based on a high likelihood of the symptoms for which the intervention would be most applicable. The reader must take this into consideration when considering the data in practice.

The skin normally provides a significant barrier to the penetration of large molecular weight molecules, however the use of magnetophoresis has been shown to significantly improve skin penetration of both small molecules and larger 20 nm gold nanoparticles [19–22]. Integration of the skin penetration technology into wearable devices, such as the knee guard, to enhance the availability of biologically active molecules over prolonged periods of time represents an entirely new therapeutic approach to the management of degenerative conditions such as OA. The participants in the present study had not received a specific diagnosis of OA and so there is considerable potential value in pursuing studies to test the value of the knee guard device in patients with a diagnosis of early stage knee OA.

A concern arising from the trial was that there was a significantly greater number of participants with skin breakdown (24% v 2%) or some form of skin irritation (51% v 0%) in the knee guard group compared to the NSAID gel group. The majority of these skin reactions were discovered on the weekly contact by the research assistant and through evaluating participant diaries. Subsequent research has further optimized the formulation to significantly reduce the risk of skin irritation.

## Conclusion

A magnetophoretic device that can administer key ground substance components through the skin over the knee joint has been shown to achieve equivalent improvements in physical function as measured by the KOOS function scale but not the AFS over 2 weeks when compared to a commercially available NSAID gel. Pain rating improvements were better with the knee guard device. This device has potential to facilitate participation in physical activity and exercise, which is an effective first line treatment for individuals at risk of developing knee OA. Caution should be exercised during the further development of this device because the device group reported substantially more skin breakdown. This pilot trial provides a basis on which to pursue further research to fully evaluate the therapeutic potential of this innovative treatment approach.

## Supporting information

S1 Checklist Consort checklist. (PDF)

S1 File Copy of trial data. (XLSX)

S2 File Trial protocol approved by ethics. (PDF)
