# Supplementary material for: A randomised pilot equivalence trial to evaluate diamagnetically enhanced transdermal delivery of key ground substance components in comparison to an established transdermal non-steroidal anti-inflammatory formulation in males with prior knee injury
Source: PLoS One. 2019 Feb 22;14(2):e0211999. doi: 10.1371/journal.pone.0211999 (PMC6386272; doi:10.1371/journal.pone.0211999)
Supplement: S2 File — (PDF) [file pone.0211999.s003.pdf]

Schedule 1 – Research Program

Aim:
To compare the equivalence of two self-treatments for knee osteoarthritis (OA) over a two week period. One of the treatments is a widely used topically applied gel (Voltaren), while the other is a magnetically delivered compound that the company has developed. The Client has preliminary evidence of safety and effects in a small group and wish now to test the equivalency to the commonly available Voltaren gel.

Milestones:

Approximate date of UQ Human Ethics approval	9 January 2015
Commencement of patient recruitment	9 January 2015
Estimated date for 50% completion of patient enrolment	20 March 2015
Estimated date for completion of patient enrolment	19 April 2015
Completion of data collection	18 May 2015
Data input and cleaning completed (database locked)	25 May 2015
Completion of data analysis	12 June 2015
Initial Draft Report due	15 June 2015
Final Report due	29 June 2015
Manuscript ready for publication	31 July 2015

Study Objectives:
1. To determine if daily use of the knee guard device will improve physical function in individuals who engage in regular exercise but have knee discomfort related to previous knee injury and surgery.
2. To determine if the improvement in physical function with the knee guard device is equivalent to the improvement produced by an established non-steroidal anti-inflammatory formulation (NSAID patch).

Primary outcome measure:
Time required to complete a battery of functional lower limb tasks.

Secondary outcome measure:
- Knee injury and osteoarthritis outcome score (KOOS).
- Lower Extremity Function Scale (LEFS).
- Pain Numerical Rating Scale (NRS).

Primary end point: Two weeks

Primary Hypotheses:
1. Utilization of the knee guard device on a daily basis over two weeks will reduce the time required to complete a battery of functional lower limb tests.
2. Utilization of the knee guard device on a daily basis over two weeks will produce an equivalent improvement in performance on the battery of functional lower limb tests to the daily administration of a NSAID patch.

Secondary Hypotheses:
3. Utilization of the knee guard patch on a daily basis over two weeks will improve:
   (a) knee injury and osteoarthritis outcome score (KOOS),
   (b) lower extremity functional scale scores (LEFS), and
   (c) pain NRS scores.
4. The improvement on KOOS, LEFS and NRS will be equivalent between the NSAID patch and knee guard patch applied daily basis over two weeks.

Background:
Osteoarthritis affects more people than any other joint disease. It is the most prevalent form of arthritis and is a major source of disability in developed countries and increasingly in developing countries (Arden and Nevitt, 2006). Knee osteoarthritis is the second most common form of osteoarthritis and the prevalence of knee pain ranges from 18 – 28% in over 55s in the United Kingdom (Arden and Nevitt, 2006). Pain is the chief symptom of most patients, prompting them to seek medical attention. Because there is no cure and disease modifying interventions are still under investigation, current treatment strategies are primarily aimed at reducing pain and improving joint function (Zhang et al., 2008). Established management strategies recommended in international treatment guidelines include both non-pharmacologic and pharmacologic modalities of therapy (Zhang et al., 2008). There is also increased use of joint replacement surgery although some patients continue to experience pain even after joint replacement (Wylde et al., 2011).

Osteoarthritis is a major source of disability in elderly individuals, compromising their quality of life and producing a significant economic burden (Guccione et al., 1994). With an aging population the prevalence and burden of this disorder is increasing rapidly.

History of injury to a joint is associated with increased risk of osteoarthritis in cross sectional studies (Davis et al., 1989) and also importantly in prospective studies with follow-up greater than 35 years (Gelber et al., 2000). Obesity is a major risk factor although knee injury may be an independent risk factor, particularly for unilateral knee osteoarthritis (Davis et al., 1989). The study by Gelber et al (2000) reported a greater than 5 fold increased risk of developing knee osteoarthritis in those with a documented history of knee injury (Gelber et al., 2000). The cohort with significant prior knee injury is therefore a key group to target for preventative interventions.

Given the steadily increasing economic and societal burden of osteoarthritis there is an increased focus on preventive strategies to minimize the risk of both the development and progression of OA. This is also of critical importance in terms of individual quality of life as individuals live longer. Strategies that might prevent the development of OA are therefore worthy of investigation and development. Such strategies may be of particular importance as a secondary preventive strategy for those individuals with a history of significant knee injury and surgery. Given that exercise is a recommended intervention to limit the impact of osteoarthritis (Zhang et al., 2008) it is important to ensure that those with previous joint injury are able to maintain a reasonable level of exercise and activity without undue discomfort.

Osteoarthritis is characterized by changes in the synovial membrane with limited synovitis, loss and erosion of articular cartilage and osteophyte formation, as well as changes in sub-chondral bone (Hochberg et al., 2013). Chondroitin sulphate and glucosamine sulphate exert a beneficial effect on the metabolism of different cell lines: chondrocytes, synovocytes and osteocytes from sub-chondral bone, all involved in the development of osteoarthritis (Hochberg et al., 2013, Henrotin and Lambert, 2013). They increase type II collagen and proteoglycan synthesis in human articular chondrocytes and reduce the production of some pro-inflammatory factors and proteases and improve the anabolic/catabolic balance of the extracellular cartilage matrix (Hochberg et al., 2013, Henrotin and Lambert, 2013).

Clinical trials have reported some beneficial effects of oral chondroitin and glucosamine on pain and function although results have been variable. Results from clinical trials in knee osteoarthritis demonstrate a small but significant reduction in the rate of decline in joint space width and some symptomatic improvement (Hochberg et al., 2013). Chondroitin and glucosamine therefore appears to offer potential benefits as a means of limiting the development of OA in at risk individuals. These benefits have been supported by the recommendations of international guidelines (Zhang et al., 2008). Concerns have been expressed however in relation to the bioavailabity of chondroitin following oral administration.

The knee guard product is a wearable knee support intended to help users maintain a healthy knee joint and enhancing their recovery from injury. It uses diamagnetic force to deliver therapeutic ingredients through the skin and into the body more effectively than topical creams or oral supplements (Benson et al., 2013). The active agents include glucosamine hydrochloride, hyaluronic acid and chondroitin sulphate in a specifically developed Lubrecin formulation which has received TGA approval. Due to the large molecular weight of the formulation components it is difficult for them to penetrate the outer layers of the skin. Using a specific formulation and patented diamagnetic repulsion technology, the product offers enhanced bioavailability of the active molecules in the tissue surrounding the knee joint.

The proposed study will assess the effect of the knee guard device and the diamagnetic technology in a cohort of individuals with prior knee injury and surgical intervention who are at increased risk of developing osteoarthritis.

Study Design:
The study will be a pilot equivalence trial (Snapinn, 2000) comparing the knee guard device to the current standard treatment in the form of a NSAID gel or patch. The primary outcomes will be improvement in lower limb function and improvement in KOOS score after 2 weeks of daily treatment. The study will be conducted in adult males aged 40–55.

Participants:

Inclusion Criteria:

- Males aged 40–55
- Prior history of knee injury requiring arthroscopic surgery and/or,
- Prolonged history of recurrent knee pain (>2 / 10 on pain numerical rating scale)
- In good general health
- Currently participating in regular physical activity (>2 hours per week).

Exclusion Criteria:

- No patellofemoral joint dysfunction
- No ligamentous deficiency
- No history of cardiac disease
- No history of high blood pressure
- No history of asthma
- No history of diabetes

Measures:
Time-Points:
Testing will be carried out at baseline and at two weeks.
The primary end point will be after 2 weeks of knee guard or NSAID use. Participants will be randomly assigned to receive either the Knee Guard treatment or topical NSAID treatment based on a pre-assigned schedule. All investigators involved in participant testing will be blind to treatment allocation. Treatment will commence on the day after baseline testing.

Trial packs will be sequentially numbered, but OBJ will randomly allocate either BG or Voltaren to the packs and will retain that randomisation schedule until the end of the trial.

Test Procedures:

Table 1 Battery of functional tests

Test	Measure
Timed Balsom agility run	seconds
Timed step test	seconds
Timed shuttle run	seconds
Stair climb (2 flights)	seconds

Table 2 Clinical outcome measures

Test	Measure
Knee injury and osteoarthritis outcome score (KOOS)	Normalized score for each sub scale and combined normalized score
Lower extremity Functional Scale	Total score/80
Pain Numerical Rating Scale (NRS)	0 no pain, 10 worst pain imaginable

Trial Endpoints:

Primary Functional Endpoint
- Total time required to complete the battery of functional tests

Primary Clinical Endpoint
- KOOS score

Secondary Clinical Endpoints
- LEFS score
- Pain NRS

Functional Measures:

Timed step test: The participant is asked to stand on a 6” block with a knee immobilizer applied to the unaffected leg. On the command “go” the subject is asked to step up and down 20 times as rapidly as possible. The sole and heel of the unaffected leg must touch the ground and the step each time. The time in seconds taken to complete 20 steps is recorded.

Time 10 m shuttle run test: A 10 metre straight is marked on the floor with cones or tape at each end. Two wooden blocks are placed at the finish line. The participant is asked to run as fast as possible from the starting line to the finishing line, pick up a block, return the block to the ground behind the starting line, run back to the finishing line, pick up the 2nd block and back to the start line. Time in seconds is recorded.

Timed Balsom agility run: A 15 metre course with 5 gates that requires two changes of direction and two turns is established. Participants are asked to run through the course as rapidly as possible and the time taken from going through the start gate to the finish gate is recorded. Time in seconds is recorded.

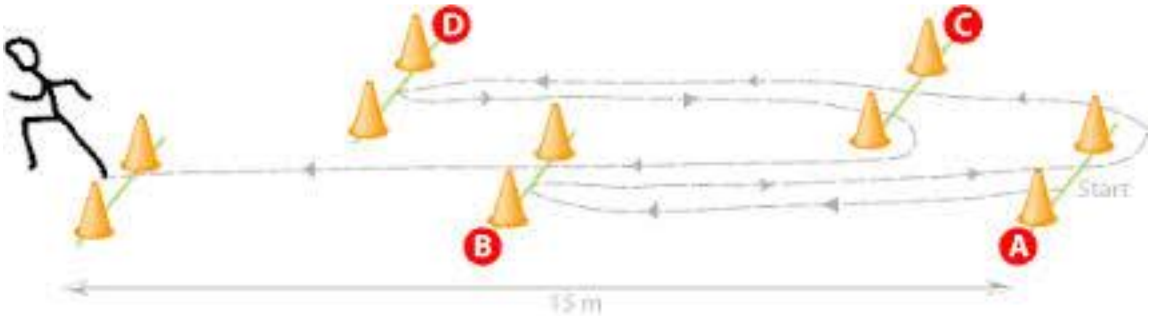

The total time required to complete all three tests will be recorded as an aggregated function score.

Self-Report Measures:

Knee Injury and Osteoarthritis outcome score (KOOS):

The KOOS is an instrument to assess a patient’s opinion about their knee and associated problems. It is intended for use with knee injury that can result in post-traumatic osteoarthritis. It consists of 5 subscales; pain, other symptoms, function in daily living, function in sport and knee related quality of life. It has high test retest reliability (ICC >0.75). The test will be administered as a paper and pencil test.

Normalized scores will be calculated for each subscale and a mean normalized score will be calculated for the overall scale.

Lower Extremity Functional Scale (LEFS):
The LEFS is a 20 item questionnaire about a person's ability to perform everyday tasks. It assesses activity limitations associated with a lower limb problem. It has high test retest reliability (ICC 0.94). The test will be administered as a paper and pencil test.

A normalized score will be calculated.

Numerical Rating Scale (NRS) for pain: the pain NRS will be an 11 point scale, with 0 being 'no pain' and 10 being 'worse pain imaginable'. Participants will be asked to rate the level of pain they are currently experiencing.

The whole number from 0 to 10 will be recorded.

Power calculation/Sample size:
A sample size of 114 participants will be required to determine a 7% equivalence rate on the KOOS with an 11% SD assuming 90% power and 95% confidence interval. A 10% drop out rate has been included within this sample size.

Study Procedure:
A group of 114 male participants will be recruited through an advertising campaign from the general community, sporting clubs and Masters sports groups in the Brisbane metropolitan area. Following initial contact from volunteers, a preliminary telephone interview will determine the participant's general suitability for the study and arrange an appropriate time for testing. Screening questionnaires will be exchanged by email. Testing will be carried out at the St Lucia campus of University of Queensland.

Baseline testing:
Participants will undergo screening to determine their suitability for inclusion in the study. This will include completing the Fitness Australia Adult Pre-Exercise Screening tool (Appendix 1). Participants who meet the inclusion criteria, have no exclusion criteria and are classified as low risk on the Pre-Exercise Screening Tool will be asked to provide written informed consent. Baseline measures of height and weight will be obtained.

Participants will then be asked to complete the KOOS and LEFS (Appendix 2 and 3).

Subsequent to completing the outcome questionnaires participants will complete the battery of functional tests. The tests will be completed in the assigned order.

On completion of the functional tests participants will be asked to complete the pain NRS to rate any pain they experienced during the functional tests.

This will complete the baseline testing.

Supply of medication:
Participants assigned to the knee guard groups will be provided with a two week supply of the knee guard device and instructed in its use for a period of 8 hours each day.

The patch contains 10mg Glucosamine, 2.5mg Chondroitin Sulphate, 2.5mg Hyaluronic Acid and 40mg Menthol in a gel formulation. Based in in vitro studies 8.8% of the glucosamine dose, 1.2% of the chondroitin dose and 0.32% of the hyaluronic acid dose is delivered through the skin.

The maximum magnetic field produced by the patch is only 2.25% of the accepted USA and EU magnetic field exposure standards.

Participants assigned to the NSAID group will be supplied with NSAID medication (Voltaren gel (diclofenac sodium topical gel) 1% and advised to use it in compliance with the published dosage requirements on a daily basis over the two week trial period.

Participants will be provided with daily diary to rate levels of pain and discomfort, any side effects or problems associated with medication or device use and to record actual use periods for the knee guard and NSAID.

Follow-up Testing:
After two weeks of knee guard or NSAID use participants will be asked to attend a second testing session in which they will complete the outcome questionnaires, functional test battery, and NRS in the same order as the baseline testing. At the end of this test session participants will also be asked to complete a short questionnaire to obtain information about their experiences with using the knee guard patch.

Monitoring:
Participants will be contacted by telephone or email on two occasions in the two week period post testing to inquire about any soreness post testing or any side effects related to the medication or device use.

Data Analysis:
Data will be managed and analysed using SPSS v21 software, with significance set at $p<0.05$.

Primary Hypotheses:
1. An initial analysis using paired t-tests will be carried out to compare the time required to complete the battery of functional tests at 2 weeks to the time required to complete the tests at baseline. Paired t-tests will also be used to compare KOOS score at 2 weeks to baseline scores.
2. Analysis of variance will be used to compare improvement in functional test time and KOOS score between the body guard and NSAID conditions.

Secondary Hypotheses:
1. Paired t-tests will be used to compare differences in LEFS scores and discomfort and pain NRS scores at 2 weeks compared to baseline.

Time-Frame & Feasibility:
It is envisaged that it will require a total period of six months (excluding the period mid-December and January), as recruitment rates to recruit and test a sufficient cohort of participants are generally very low at this time.

Ethical Issues:
There are no major ethical issues associated with this study. Participants will be healthy volunteers. Participants will be adequately screened prior to participating in the exercise testing. They will be monitored throughout testing and for any adverse reactions to the medications. Full confidentiality of information will be maintained. Participants will be paid $50 for the two testing sessions to cover their expenses related to participating in the study. All treatments will be supplied free-of-charge.

References:

ARDEN, N. & NEVITT, M. C. 2006. Osteoarthritis: epidemiology. *Best Pract Res Clin Rheumatol,* 20**,** 3-25.

BENSON, H. A. E., MCILDOWIE, M. & PROW, T. 2013. Magnetophoresis: skin penetration enhancement by a magnetic field. *In:* DRAGICEVIC-CURIC, N. & MAIBACH, H. (eds.) *Chemical Methods in Penetration Enhancement.* Springer (In Press).

DAVIS, M. A., ETTINGER, W. H., NEUHAUS, J. M., CHO, S. A. & HAUCK, W. W. 1989. The association of knee injury and obesity with unilateral and bilateral osteoarthritis of the knee. *Am J Epidemiol,* 130**,** 278-88.

GELBER, A. C., HOCHBERG, M. C., MEAD, L. A., WANG, N. Y., WIGLEY, F. M. & KLAG, M. J. 2000. Joint injury in young adults and risk for subsequent knee and hip osteoarthritis. *Ann Intern Med,* 133**,** 321-8.

GUCCIONE, A. A., FELSON, D. T., ANDERSON, J. J., ANTHONY, J. M., ZHANG, Y., WILSON, P. W., KELLY-HAYES, M., WOLF, P. A., KREGER, B. E. & KANNEL, W. B. 1994. The effects of specific medical conditions on the functional limitations of elders in the Framingham Study. *Am J Public Health,* 84**,** 351-8.

HENROTIN, Y. & LAMBERT, C. 2013. Chondroitin and glucosamine in the management of osteoarthritis: an update. *Curr Rheumatol Rep,* 15**,** 361.

HOCHBERG, M., CHEVALIER, X., HENROTIN, Y., HUNTER, D. J. & UEBELHART, D. 2013. Symptom and structure modification in osteoarthritis with pharmaceutical-grade chondroitin sulfate: what's the evidence? *Curr Med Res Opin,* 29**,** 259-67.

SNAPINN, S.M. 2000. Noninferiority trials. *Curr Control Trials Cardiovasc Med*, 1, 19-21.

WYLDE, V., HEWLETT, S., LEARMONTH, I. D. & DIEPPE, P. 2011. Persistent pain after joint replacement: prevalence, sensory qualities, and postoperative determinants. *Pain,* 152**,** 566-72.

ZHANG, W., MOSKOWITZ, R. W., NUKI, G., ABRAMSON, S., ALTMAN, R. D., ARDEN, N., BIERMA-ZEINSTRA, S., BRANDT, K. D., CROFT, P., DOHERTY, M., DOUGADOS, M., HOCHBERG, M., HUNTER, D. J., KWOH, K., LOHMANDER, L. S. & TUGWELL, P. 2008. OARSI recommendations for the management of hip and knee osteoarthritis, Part II: OARSI evidence-based, expert consensus guidelines. *Osteoarthritis Cartilage,* 16**,** 137-62.
